# Long-term observation of the egg and chick size in the nests of *Larus ichthyaetus* in Lake Chany, Russia

**DOI:** 10.1038/s41597-022-01454-8

**Published:** 2022-06-29

**Authors:** Alexander K. Yurlov, Natalia I. Yurlova, Maria Yu. Garyushkina, Marina A. Selivanova, Hideyuki Doi

**Affiliations:** 1 Institute of Systematics and Ecology of Animals, Siberian Branch of Russian Academy of Sciences, Frunze Str., 11, Novosibirsk, 630091 Russia; 2 Graduate School of Information Science, University of Hyogo, 7-1-28 Minatojima-minamimachi, Chuo-ku, Kobe, 650-0047 Japan

**Keywords:** Animal migration, Conservation biology

## Abstract

This data set describes the long-term observation and morphological study of the eggs of the great black-headed gull *Larus ichthyaetus* in the gull nesting colonies on the islands of Lake Chany. Lake Chany is located in the Baraba forest-steppe of the West Siberian Plain, Russia, between the Ob and Irtish rivers. Lake Chany is protected by the Ramsar Convention on the Wetlands of International Importance, indicating that the lake is an important site for migratory birds, including *L. ichthyaetus*. This dataset contains the size and fate of all eggs, as well as the size of hatched chicks in 1164 observed *L. ichthyaetus* nests from 1993 to 2003.

## Abstract (summary)

**Table:** 

Measurement(s)	size
Technology Type(s)	Observation
Factor Type(s)	egg and chick size
Sample Characteristic - Organism	Larus ichthyaetus
Sample Characteristic - Environment	lake
Sample Characteristic - Location	Russia

## Background & Summary

Lake Chany is located in the Baraba forest-steppe of the West Siberian Plain, Russia, between the Ob and Irtish Rivers (54°30′–55°09′ N, 76°48′–78°12′ E). The Lake Chany basin is among the largest in Russia, and the lake itself has the greatest water surface in West Siberia^1^. The waters, coastal zones, and islands of Lake Chany are favorable for breeding, molting, and feeding of more than 280 species of birds. In spring and autumn, numerous migratory waterbirds (over 300 000), such as swans, geese, ducks, coots, waders, gulls, terns, cranes, stop at the lake during migration^1,2^. Some of the species are rare and endangered and are listed in the Red Data Book of Russia and the Red List of the International Union for Conservation of Nature and Natural Resources (IUCN)^3^. Lake Chany is protected by the Ramsar Convention on Wetlands of International Importance, indicating that the lake is an important site for migratory birds, including *L. ichthyaetus*. Currently, most of the great black-headed gull colonies are in a vulnerable state; therefore, this species is recorded in the Red Data Books of the Russian Federation and Kazakhstan^4,5^. Therefore, it is important to observe the dynamics of their reproduction. Inter-annual changes in the onset of egg-laying and morphological parameters of eggs were pronounced during the years of our research and were probably determined by the course of spring and feeding conditions^6,7^. Egg size may be affected by food availability^8,9^. Some studies suggested a relationship between egg size and morphological traits, survival, and growth rate of chicks hatching from them [reviewed in^10,11^].

Here, we provide eight years of raw field data (1993, 1994, 1996–1998, 2001–2003) from our egg and chick morphological study on great black-headed gull *Larus ichthyaetus* breeding on islands of Lake Chany, Russia^12^.

## Methods

We surveyed three islands of Lake Chany: Uzkoredkii (54° 58′ 15′′ N, 77°27′04′′ E), Reden’kii (54° 56′ 05′′ N, 77° 22′ 27′′ 52 E), Korablik (54° 59′ 31′′ N, 77° 40′ 38′′ E). The studied intertidal habitats are rarely reached by humans.

Gull nests were counted in colonies by regular surveys over eight years (1993, 1994, 1996–1998, 2001–2003) on the islands of Lake Chany. Colonies were visited daily or sometimes every other day. To minimize the disturbance caused by the investigation, the time spent working, within view of the gulls was restricted to a maximum of forty minutes per study plots. We noted nest content at every visit for the presence of eggs or chicks. In total, there were 1 164 nests under observation. Nests contained 1 (n = 140), 2 (n = 518), 3 (n = 504) or 4 (n = 2) eggs. Modal clutch size of the great black-headed gull is two or three eggs, varying seasonally. The length and width of the eggs were measured using Vernier calipers (division accuracy 0,1 mm) and numbered with a waterproof marker. Egg volumes were estimated using Hoyt’s equation: Volume = 0.51 * Length * Width * Width/1000^13^. We determined the volume of 2117 great black-headed gull eggs.

As the laying of eggs has already started by the first visit to the colony, the date of the beginning of egg laying was calculated by subtracting the average length of the incubation period of great black-headed gulls (27 days) from the hatching date of first chick in the nest (n = 559 nests). If the hatching date was not known, the clutch initiation date was determined by subtracting the number of days of incubation from the date that the nest was first discovered (n = 469 nests). The stage of incubation was estimated from the change in position of an incubated egg placed in water^14,15^. The technique’s accuracy varied throughout incubation and mean prediction error fall between 0–4 days. On average, egg flotation estimated an embryo’s developmental age to within 1.9 ± 1.6 days (mean ± 1 SD)^16^. Only 47 nests were found during egg laying. Great black-headed gulls usually laid eggs at intervals of two days. Incubation started as soon as the first egg was laid, so eggs hatched asynchronously, one or two days apart.

Whenever possible, we determined the within-clutch laying sequence of eggs (1^st^, 2^nd^, 3^rd^, and 4^th^). A complete laying sequence was established by observation in 47 cases. In about 48% of clutches the position in laying sequence was established on the basis of the sequence of hatching. In other cases, if we could distinguish within-clutch distinct flotation levels of eggs, we numbered eggs according to the stage of incubation. Sometimes this technique for distinguishing egg laying order were used in other seabirds^17,18^.

We recorded the pipping date (i.e. appearance of star-like bursts) and the actual hatching date of the individual eggs. Wet chicks were registered as hatchlings of that day; dry chicks were registered as 1 day old. Chicks older than two days left the nest and moved to a location nearby. Newly hatched gull chicks were captured by hand at nests, ringed, and measured. We determined wing, tarsus, and head length using a ruler with zero-stop and vernier calipers and body weight measured using Pesola spring balances for 747 chicks of great black-headed gulls, and 457 of them hatched from eggs that were measured.

## Data Records

The all data were deposited in Zenodo 10.5281/zenodo.5175551^12^. The data name, unit and method for the data were described in Table 1.

**Table 1:** Data name, unit and method for the data.

Data	unit	Summary of methods
Date of detection (observation start date)	date	
Date of detection (1=1 January)	days of year	
Date of clutch initiation	date	
Date of clutch initiation (1=1 January)	days of year	
Number of eggs in the clutch	number	Dr. Yurlov counted
Number of chicks hatched in the nest	number	Dr. Yurlov counted
Method of determining egg sequence (1st, 2nd, 3rd): D — direct observation, F — according egg floatation test, H — according to hatching order		Egg rank or within-clutch laying sequence of eggs (1st, 2nd, 3rd) was estimated for nests that were visited during laying (D — direct observation), for nests, in which the stage of incubation of eggs was estimated by floatation test (F), and for nests with known the order of hatching of each egg (H). In the rest of the nests, the eggs were numbered randomly (NA).
Length of egg (mm)	mm	Maximum length of the egg measured with calipers (division accuracy 0,1 mm)
Width of egg (mm)	mm	Maximum width of the egg measured with calipers (division accuracy 0,1 mm)
Volume of egg (cm^3^)	cm^3^	Volume of egg calculated using the formula: Volume = 0,51 * Length*Width*Width/1000
Eggshell coloration of the egg		Dr. Yurlov visually determined the color of eggshell.
Fate of the egg	category	Dr. Yurlov observed the fate of egg
Ringing date of nestling	date	Date for ringing of the chick
Ringing date of nestling (1=1 January)	days of year	Date for ringing of the chick
Hatching date of nestling	date	Date for the chick hatched
Hatching date of nestling (1=1 January)	days of year	Date for the chick hatched
Age of chick at ringing (days)	days	Age of chick at ringing
Ring number on the chick	number	Chicks ringed with aluminum and steel rings with identification number “C”, “DB”, “DS” series
Wing length of chick, mm	mm	Maximum wing length of the chick - maximum distance between the carpal joint and the tip of wing measured with a ruler with a zero-stop
Tarsus length of chick, mm	mm	Maximum tarsus length of the chick measured with calipers (division accuracy 0,1 mm)
Bill length 1 of chick, mm	mm	Distance between the base of the skull and tip of the upper mandible measured with calipers (division accuracy 0,1 mm)
Bill length 2 of chick, mm	mm	Distance between the posterior end of nostrils and tip of the upper mandible measured with calipers (division accuracy 0,1 mm)
Head length of chick, mm	mm	Maximum head length of the chick - maximum distance between the occipital end of the head and the tip of the bill measured with calipers (division accuracy 0,1 mm)
Body weight of chick, g	g	Total body weight measured using Pesola spring balances

## Technical Validation

The long-term monitoring program for was the egg and chick size in the nests of *Larus ichthyaetus* designed by A.K.Y. A.K.Y. identified the nest and measured the values which we provided in the database. Then A.K.Y. recorded all data in an Excel file. deceased before preparing the data base and this paper. The other authors carefully prepared the data sheet with double checking by the authors.

## Data Availability

We did not use the custom code for the data management.

## References

[1] Yurlov, A. K. *et al*. Vertebrates of Chany Lake and surrounded area. Pages 162–203 in O.F. Vasilyev, J. Veen editors. *Chany Lake (West Siberia) environmental profile*. Academic Publishing House GEO, Novosibirsk 2015.

[2] Veen, J. *et al*. *An atlas of movements of Southwest Siberian waterbirds*. Wetlands International, Wageningen, The Netherlands 2005.

[3] IUCN. The IUCN Red List of Threatened Species. Version 2021-2. https://www.iucnredlist.org 2021.

[4] Danilov-Danilyan, V. I. (ed.). Red Data Book of Russian Federation. *Animals*. Astrel, Moscow 2001.

[5] The Red List of Republic of Kazakhstan. http://www.redbookkz.info.

[6] Brommer JE, Rattiste K, Wilson AJ (2008). Exploring plasticity in the wild: laying date-temperature reaction norms in the common gull Larus canus. Proceedings of the Royal Society B: Biological Sciences.

[7] Votier SC, Hatchwell BJ, Mears M, Birkhead TR (2009). Changes in the timing of egg-laying of a colonial seabird in relation to population size and environmental conditions. Marine Ecology Progress Series.

[8] Reid WV (1987). Constraints on clutch size in the Glaucous-winged Gull. Studies in Avian Biology.

[9] Salzer DW, Larkin GJ (1990). Impact of courtship feeding on clutch and third-egg size in Glaucous-winged Gulls. Animal Behaviour.

[10] Williams TD (1994). Intraspecific variation in egg size and egg composition in birds: effects on offspring fitness. Biological Reviews.

[11] Krist M (2010). Egg size and offspring quality: a meta-analysis in birds. Biological reviews.

[12] Yurlov AK, Yurlova NI, Garyushkina MY, Selivanova MA, Doi H (2022). Zenodo.

[13] Hoyt DF (1979). Practical methods of estimating volume and fresh weight of bird eggs. Auk.

[14] Westerskov K (1950). Methods for determining the age of game bird eggs. Journal of Wildlife Management.

[15] Van Paassen AG, Veldman DH, Beintema AJ (1984). A simple device for determination of incubation stages in eggs. Wildfowl.

[16] Ackerman JT, Eagles-Smith CA (2010). Accuracy of egg flotation throughout incubation to determine embryo age and incubation day in waterbird nests. The Condor.

[17] Nisbet ICT (1975). Selective effects of predation in a tern colony. The Condor.

[18] Gochfeld M (1977). Intraclutch egg variation: the uniqueness of the common tern’s third egg. Bird-Banding.

